# Transcranial Direct Current Stimulation: Considerations for Research in Adolescent Depression

**DOI:** 10.3389/fpsyt.2017.00091

**Published:** 2017-06-07

**Authors:** Jonathan C. Lee, Charles P. Lewis, Zafiris J. Daskalakis, Paul E. Croarkin

**Affiliations:** ^1^Temerty Centre for Therapeutic Brain Intervention, Campbell Family Mental Health Research Institute, Centre for Addiction and Mental Health, Toronto, ON, Canada; ^2^Faculty of Medicine, Department of Psychiatry, University of Toronto, Toronto, ON, Canada; ^3^Mayo Clinic Depression Center, Department of Psychiatry and Psychology, Mayo Clinic, Rochester, MN, United States

**Keywords:** adolescent depression, neurostimulation, non-invasive brain stimulation, transcranial current stimulation, transcranial direct current stimulation

## Abstract

Adolescent depression is a prevalent disorder with substantial morbidity and mortality. Current treatment interventions do not target relevant pathophysiology and are frequently ineffective, thereby leading to a substantial burden for individuals, families, and society. During adolescence, the prefrontal cortex undergoes extensive structural and functional changes. Recent work suggests that frontolimbic development in depressed adolescents is delayed or aberrant. The judicious application of non-invasive brain stimulation techniques to the prefrontal cortex may present a promising opportunity for durable interventions in adolescent depression. Transcranial direct current stimulation (tDCS) applies a low-intensity, continuous current that alters cortical excitability. While this modality does not elicit action potentials, it is thought to manipulate neuronal activity and neuroplasticity. Specifically, tDCS may modulate *N*-methyl-d-aspartate receptors and L-type voltage-gated calcium channels and effect changes through long-term potentiation or long-term depression-like mechanisms. This mini-review considers the neurobiological rationale for developing tDCS protocols in adolescent depression, reviews existing work in adult mood disorders, surveys the existing tDCS literature in adolescent populations, reviews safety studies, and discusses distinct ethical considerations in work with adolescents.

## Introduction

Transcranial direct current stimulation (tDCS) is a form of non-invasive brain stimulation (NIBS) that has demonstrated efficacy in treating depression (1). Although several authors have reviewed tDCS applications in pediatric patients with neuropsychiatric disorders (2–6), herein we review evidence supporting tDCS as a putative alternative to existing treatments for depressed adolescents.

## Adolescent Depression and Unmet Needs

Up to one fifth of adolescents may experience major depressive disorder (MDD) before adulthood (7–9). Depressed adolescents experience comorbid psychiatric disorders (10) and recurrences (11), which compound health-care costs and contribute to considerable psychosocial impairment. Adolescent MDD can impact psychosocial development, education, and employability (12), costing society billions of dollars each year (13). Effective treatment options, however, remain limited.

Existing treatments for adolescent MDD, including selective serotonin reuptake inhibitors (SSRIs) and psychotherapy (14, 15), are only marginally effective (16, 17). Treatment adherence in this population is typically poor (18, 19) and limited by side effects (20). Moreover, ongoing controversies regarding the safety and efficacy of antidepressants in adolescents raise concern in clinicians and parents alike (21, 22).

## Neurobiology of Adolescent Depression

Prior work has sought to characterize the neurobiology of depression across the lifespan (23, 24) with the goal of identifying biological treatment targets (25). Studies thus far have revealed differences in the neuronal structure and chemical pathways among depressed adolescents compared to healthy controls. Observed differences include heightened activity in the amygdala during facial-emotion recognition tasks (26) and greater connectivity between the amygdala and subgenual anterior cingulate cortex (ACC) (27). Functional imaging studies have revealed altered medial prefrontal cortical connectivity with brain regions involved in executive functioning, emotion regulation, attention, and reward-based decision making (28). These differences from healthy controls may resolve with psychopharmacological intervention (29).

One proton magnetic resonance spectroscopy (^1^H-MRS) study revealed decreased gamma-aminobutyric acid (GABA) levels in the ACC of depressed adolescents compared with healthy controls (30). GABA is an inhibitory neurotransmitter that plays a key role in the neurocircuitry of reward which, when dysregulated, may be linked to anhedonia. Another ^1^H-MRS study showed dysregulation in ratios of *N*-acetyl aspartate to creatine and choline to creatine in the dorsolateral prefrontal white matter (31). Other ^1^H-MRS studies of adolescents with treatment-resistant depression have shown elevated choline levels in the dorsolateral prefrontal gray matter (32, 33). Collectively, these disruptions may reflect decreased neuronal density and myelination, which may correlate with cognitive impairments commonly observed in depressed adolescents (34, 35). Despite these advances, however, the treatment of adolescent depression is most often a trial-and-error endeavor (25), and the feasibility of monitoring therapeutic response with biomarkers in clinical practice remains low (36).

Anodal tDCS is thought to be neuromodulatory, raising tonic excitation toward threshold levels, increasing the possibility that neurons will fire. Conversely, cathodal stimulation is thought to decrease cortical excitability (37, 38).

## Efficacy in Adult MDD

The history of tDCS in psychiatry dates back to the late nineteenth century, when Tigges and Arndt first documented its antidepressant and antipsychotic properties (39, 40). With the reemergence of tDCS in the modern era (41), researchers have sought to develop this portable, low-cost treatment as a frontline intervention for depression (42). Table 1 provides an overview of sham-controlled trials that suggest tDCS is efficacious in mitigating symptoms of adult depression (1, 43–45). However, some experts have argued that these individual trials lack statistical power to detect therapeutic benefit (37), and others have questioned its clinical utility altogether (46, 47). For example, Bennabi et al. (46) found no difference between sham and active tDCS, but noted a subgroup of individuals in the active tDCS arm who experienced significant clinical improvement. The authors suggest that as yet unknown factors may contribute to, or detract from, tDCS response. Variations in stimulation parameters and duration of treatment, along with different degrees of treatment resistance and the presence or absence of concomitant medication therapy, complicate interpretation of the data. Some meta-analyses have found no beneficial effect (48), while others have shown that tDCS is efficacious in adult depression (49–51).

**Table 1:** **Summary of sham-controlled clinical trials in adult major depressive disorder (MDD) reviewed**.

Reference	*N*	AD use	Stimulation parameters	Measures	Outcome	Adverse effects
Fregni et al. (44)	10	Unknown	(1) Anodal L-DLPFC (cathode RSO); 1 mA; 20 min/day for five sessions (2) Sham stimulation L-DLPFC	HRSD	Four treatment responders in active group, no treatment responders in sham group; *p* < 0.05 for both HRSD and BDI when baseline compared to scores at treatment end	All patients tolerated tDCS without complication, tDCS reported as “painless”
				BDI		

Boggio et al. (43)	40	No	(1) Anodal L-DLPFC (cathode RSO) 2 mA, 20 min/day, 10 sessions (2) Anodal left occipital cortex (3) Sham stimulation L-DLPFC	HRSD	Significant difference in HDRS when active compared to sham (*p* = 0.0018); significant difference in BDI at day 10 (*p* = 0.0045); differences persisted at 30 days post-treatment	Well-tolerated, adverse effects equally distributed across groups (*p* = 0.95), including mild transient headache, itching, mild transient redness
				BDI		

Loo et al. (45)	40	Yes	(1) Anodal L-DLPFC (cathode RSO) 1 mA, 20 min/day, five sessions	MADRS	Significant differences from baseline measures on mood questionnaires, however, no significant differences between active and sham treatments —authors attribute to concurrent antidepressant medication	No changes on neuropsychological measures (RAVLT, Trail Making, Digit Span, COWAT). Mild to moderate skin redness, itchiness (*n* = 13), tingling (*n* = 6), mild headache (*n* = 8), lightheadedness (*n* = 4), ringing in the ears (*n* = 3), blurred vision (*n* = 2), brighter or illuminated vision (*n* = 2)
				HRSD		
				BDI		

Brunoni et al. (1)	103	Yes	(1) Anodal L-DLPFC (cathode RSO) 1 mA, 20 min/day, five sessions	MADRS	Combination treatment with sertraline 50 mg and tDCS outperformed sertraline alone (*p* = 0.002) and tDCS alone (*p* = 0.03) and sham (*p* < 0.001). Factorial analysis suggested that tDCS and sertraline effects were additive	No cognitive changes. There were five cases of hypomania, and two cases of mania. The two manic episodes occurred in the combined group. Common adverse events did not differ among treatment groups (*p* = 0.17 Fisher exact test), except for redness which was more common in tDCS (*p* = 0.03)
				HRSD		
				BDI		

*AD, antidepressant; L-DLPFC, left dorsolateral prefrontal cortex; RSO, right supraorbital; HDRS, Hamilton Depression Rating Scale; BDI, Beck depression inventory; MADRS, Montgomery–Asberg depression Rating Scale; RAVLT, Rey Auditory Verbal Learning Task; COWAT, Controlled Oral Word Association Task*.

## Safety in Adults

Bikson and colleagues (52) published an extensive review on the safety literature available to date. Based on their review of current stimulation protocols and computer modeling studies, tDCS was considered safe. No serious adverse events were associated with its administration.

In a systematic review by Brunoni et al. (53), common side effects associated with tDCS—including headache, as well as localized itching, tingling, burning sensations, and discomfort—occurred at similar rates in active and sham stimulation. To date, tDCS has not been shown to induce neuronal damage. Nitsche et al. (54) did not find any elevations in neuronal specific enolase, an indicator of neuronal damage, after a standard course of tDCS. Liebtanz et al. (55) found that a current density of 142.9 A/m^2^ delivered over more than 10 min was necessary to induce brain damage in rats. This current density is multiple orders of magnitude greater than the 0.096 A/m^2^ typically delivered to the cortex in human studies (52).

## Efficacy in Children and Adolescents

Although studies of adolescent depression are lacking, researchers have applied tDCS to other pediatric psychiatric conditions. The results of these studies are summarized in Table 2. The methodologies of existing studies, including aims and design, as well as tDCS parameters such as current, electrode placement, treatment duration, frequency, and number of sessions, have varied considerably (3).

**Table 2:** **Summary of tDCS trials reviewed in child and adolescent psychiatry**.

Reference	*N*	Age	Stimulation parameters	Measures	Outcome	Adverse effects
Amatachaya et al. (56)—evaluated efficacy in ASD	20	5–8	(1) Anodal left DLPFC (1 mA, 20 min; 5 consecutive days), cathode on right shoulder (2) Sham stimulation DLPFC	CARS	At 1-week post-treatment, CARS, ATEC, and CGAS were significantly improved from baseline (*p* < 0.001, *p* < 0.001, *p* = 0.042, respectively) suggesting improved ASD symptoms	No adverse events were reported by parents/participants in sham or active sessions of repeated stimulation; three participants (same sample) reported transient erythematous rash following stimulation in a follow-up single-session study (62)
				ATEC		
				CGAS		

Andrade et al. (57)—evaluated feasibility in language disorders	14	5–12	(1) Anodal left Broca’s area (2 mA, 30 min, 10 sessions over 2 weeks), cathode RSO	Parental reports on PGI-I	tDCS considered feasible and tolerable in children, though authors recommended studies about plasticity and cognitive changes in children to confirm safety	Participants reported tingling (28.6%), itching (28.6%), acute mood changes (42.9%), irritability (35.7%), headache (14.3%), burning sensation (14.3%), sleepiness (14.3%), and trouble concentrating (14.3%) among other side effects
						Burning sensation, scalp pain, and redness were reported as “definitively related” to tDCS, while headache, sleepiness, and trouble concentrating were considered “possibly” or “probably” related to stimulation in 50% of cases
						Acute mood change and irritability were reported as unrelated to stimulation in 66% and 40% of patients

D’Urso et al. (58)—evaluated safety, efficacy, and feasibility in abnormal behaviors in ASD	12	18–26	(1) Cathodal left DLPFC (1.5 mA, 20 min, 10 sessions), anode on lateral aspect of patient’s right arm	ABC	Decrease in ABC total score (mean difference −21.7, *p* = 0.002), irritability/aggression subscale (mean difference −5.5, *p* = 0.003), social withdrawal/lethargy (−4.3, *p* = 0.03), and hyperactivity/non-compliance subscales (mean difference: 9.0, *p* = 0.002)	Two out of 10 subjects were unable to tolerate treatment procedures, but the authors did not elaborate. No adverse effects reported apart from some temporary skin irritation at the stimulation site

Schneider and Hopp (59)—evaluated efficacy in minimally verbal children with ASD	10	6–21	(1) Anodal left DLPFC (2 mA, 30 min, once), cathode RSO	BAT	Large change in pre- and post-tDCS BAT scores (*d* = 2.78, *p* < 0.0005) indicating improved syntax acquisition	None reported

Prehn-Kristensen et al. (60)—evaluated efficacy of toDCS in ADHD	12	10–14	(1) Bilateral DLPFC 0.25 mA, oscillating at 0.75 Hz, five 5-min stimulation sessions with a 1-min ISI; reference electrodes at bilateral mastoids	“Memory” computer game	Memory consolidation higher in stimulation condition (*p* = 0.004); enhanced slow oscillation sleep	Unreported

Mattai et al. (61)—evaluated feasibility and tolerability in COS	13	10–17	(1) Bilateral anodal DLPFC stimulation (2 mA, 20 min, 10 sessions), reference electrode on non-dominant forearm (2) Bilateral cathodal STG stimulation (2 mA, 20 min, 10 sessions), reference electrode on non-dominant forearm (3) Sham stimulation DLPFC or STG	Feasibility, tolerability	tDCS well tolerated in adolescent population with COS	Well tolerated, no serious adverse events occurring. Stimulation was associated with tingling (37.5%) and itching (50%)—but not significantly different from sham stimulation. Some transient redness at the stimulation site. There were no changes in mood, MMSE, MRI, ECG, or EEG. No subjects required medication changes or additional medical intervention; one subject withdrew for reasons unrelated to stimulation

*ASD, autism spectrum disorder; DLPFC, dorsolateral prefrontal cortex; CARS, Childhood Autism Rating Scale; ATEC, Autism Treatment Evaluation Checklist; CGAS, Children’s Global Assessment Scale; RSO, right supraorbital region; PGI-I, Patient Global Impression of Improvement; ABC, Aberrant Behavior Checklist; BAT, Bilingual Aphasia Test; ADHD, attention-deficit/hyperactivity disorder; toDCS, transcranial oscillatory direct current stimulation; ISI, interstimulus interval; STG, superior temporal gyrus; COS, childhood onset schizophrenia; MMSE, Mini-Mental State Examination*.

The most extensively studied pediatric population to date is children and adolescents with autism spectrum disorders (ASDs), a heterogeneous neurodevelopmental condition affecting communication, social functioning, and repetitive/stereotypical behaviors. In an open-label study of minimally verbal individuals with ASD (aged 6–21 years), Schneider and Hopp (59) examined the impact of a single session of anodal tDCS (2 mA) to the left dorsolateral prefrontal cortex (DLPFC) on vocabulary and syntax acquisition. Following the 30-min treatment session, participants demonstrated significant improvement in vocabulary and syntax scores of the Bilingual Aphasia Test, with a substantial effect size for syntax acquisition (Cohen’s *d* = 2.78). The authors reported no adverse effects from their intervention (59). Amatachaya and colleagues performed two studies with the same sample of 20 male ASD patients aged 5–8 years, both of which followed a double-blinded, sham-controlled crossover design. In the first study (56), participants underwent five sessions of anodal tDCS (1 mA, 20 min) to the left DLPFC or sham stimulation over one week, followed by a 4-week washout period, and then five sessions of the opposite condition. Researchers completed assessments of ASD symptoms at baseline and 7 days after each treatment phase. Active tDCS resulted in significant reductions in overall ASD symptom severity as well as improvements in social, sensory and cognitive awareness, and health and behavioral problem subscales. No such changes occurred with sham stimulation. In a follow-up single-session study (62), the researchers obtained resting state electroencephalography (EEG) at baseline and 24, 48, and 72 h after stimulation. Active tDCS induced increases in peak alpha frequency (PAF) at 0 and 24 h at the treatment site. Conversely, sham stimulation produced no such changes. At one week post-treatment, ASD symptom total scores, and social and health/behavioral problem subscale scores, were significantly lower in those who received active tDCS than in those who received sham. Regression analyses demonstrated significant associations between change in PAF and improvement in measures of ASD symptoms. The authors noted only mild, transient erythematous rash as the sole adverse event.

In a heterogeneous sample of children, Andrade and colleagues (57) conducted a study of the feasibility and tolerability of tDCS. Fourteen participants aged 5–12 years with learning disorders and a variety of comorbidities [which included pervasive developmental disorders/ASD, attention-deficit/hyperactivity disorder (ADHD), and intellectual disability] underwent ten 30-min sessions over two weeks of open-label anodal tDCS (2 mA) applied to Broca’s area during social and speech-related activities. Mood changes (42.9%) and irritability (35.7%) were the most common adverse effects, although participants’ parents did not uniformly attribute these to tDCS. Otherwise, the majority of other side effects (e.g., headache, itching, burning sensation/tingling, and localized erythema) were mild and transient. D’Urso and colleagues (58) also investigated the use of tDCS in an open-label study of adolescents and young adults (aged 18–26) with ASD and intellectual disability; approximately half also had a language disorder. In contrast to other studies, the authors utilized 1.5 mA cathodal stimulation to the left DLPFC for ten 20-min sessions over two weeks, with the aim of restoring inhibitory function and reducing behavioral symptoms. Significant reductions in the Aberrant Behavior Checklist total score and several subscale scores (irritability, social withdrawal, and hyperactivity) were observed between baseline assessment and reassessment one week after tDCS. Minor, transient skin irritation was the only noted adverse effect.

One study to date has examined the effects of tDCS specifically in children and adolescents with ADHD. In a sample of 12 male patients (aged 10–14), Prehn-Kristensen and colleagues (60) investigated whether transcranial oscillatory direct current stimulation (toDCS) improved memory consolidation during slow-wave sleep. Utilizing a double-blind, sham-controlled crossover design, the authors administered a single session of anodal toDCS (0.25 mA, oscillating at 0.75 Hz, five 5-min stimulation sessions with a 1-min interstimulus interval) to the DLPFC bilaterally during slow-wave sleep (determined by EEG). Performance on a declarative memory task was measured before and after stimulation. Active toDCS enhanced slow oscillations on EEG and improved declarative memory performance to a level comparable with a comparator group of 12 healthy control children, while sham toDCS did not result in enhanced slow oscillations or improvement in sleep-dependent memory consolidation.

A single study examined tDCS in youth with schizophrenia. Mattai and colleagues (61) conducted a double-blinded, sham-controlled tolerability study in a sample of 13 patients, aged 10–17, with early-onset schizophrenia. Notably, all patients in the study were receiving concurrent treatment with clozapine. Some participants were also prescribed other psychotropic medications. Participants received either active or sham anodal tDCS (2 mA) to the DLPFC bilaterally, or active or sham cathodal tDCS (2 mA) to the superior temporal gyrus bilaterally. Patients received ten 20-min sessions over two weeks, and those undergoing sham treatment were eligible for additional ten sessions of active tDCS. Tingling sensations, itching, and fatigue were the most common side effects reported, with no difference in rates between active tDCS and sham groups. No serious adverse effects occurred during the 4- or 6-week participation period. Additionally, neither sham nor active tDCS induced significant changes on structural head MRI, EEG, or electrocardiogram (ECG). This study was not powered to evaluate tDCS efficacy.

## Concurrent and State-Dependent Interventions

While much prior research has examined the effects of tDCS on cognitive, neurophysiologic, or symptomatic measures in neuropsychiatric disorders, some studies have investigated the use of tDCS in conjunction with motor and cognitive tasks as well as therapeutic activities. The interaction between tDCS and concurrent tasks is complex and incompletely understood.

The timing of tDCS delivery in relation to a cognitive or motor task—before, during, or after the task—appears to influence its effects. Stimulation occurring before, but not during or after, motor training increases cortical excitability as measured by transcranial magnetic stimulation (TMS) (63). However, there is also evidence that cognitive (64) and motor (65) tasks administered after tDCS can partially reverse or abolish anodal effects on cortical excitability. Moreover, tDCS-induced excitability changes differ between cognitive and motor tasks performed during anodal and cathodal stimulation (66). The cumulative effects of stimulation paired with tasks may be significant as well; a single session of tDCS with substance-related cues demonstrated opposite effects on event-related potentials compared to repeated sessions of tDCS (67). The specific characteristics of the concurrent task also may determine the effects of the stimulation–task interaction. In a motor learning task, the speed of the task during concurrent tDCS determined whether learning was facilitated or inhibited (68). Active movement in a motor task during stimulation had different effects on excitability than did passive movement (69). Such task-specific effects are not limited to concurrent motor activities; the degree of cognitive demand of an executive functioning task during stimulation affected performance (70).

Researchers have also evaluated more cognitively complex and clinically relevant concurrent tasks. tDCS administered prior to addiction-related cues reduced cravings and substance use (71, 72). By contrast, tDCS administered concurrently with a craving-inducing cue task increased cravings during stimulation but decreased cravings at rest (73). One potential advantage of tDCS is the possibility of enhancing another concurrent therapeutic activity, including psychotherapy, cognitive remediation, skill training, or other modalities. Moreover, its low cost and portability may enable patients to utilize tDCS, with close clinical monitoring, in their homes or other naturalistic settings where such therapeutic activities occur, thereby enhancing the accessibility of tDCS and integrating it into existing intervention plans (38). Although the literature is sparse, there are emerging reports of the use of tDCS concurrently with therapeutic interventions such as integrative speech therapy (74) and aerobic exercise (75) in human patients. However, much remains to be understood regarding the specifics of timing, repetition, and task-specific parameters to maximize the therapeutic effects of concurrent interventions.

## Safety in Children and Adolescents

Transcranial direct current stimulation is considered safe in adults (52), and preliminary evidence from studies with child and adolescent patients suggests a similar safety profile (2). Unlike antidepressant medications, tDCS is not associated with sexual side effects, serotonin syndrome, or suicidality. Krishnan and colleagues (2) found tingling, itching, redness, and scalp discomfort to be the most common side effects in their systematic review of adolescent tDCS trials. These effects were self-limited, lasting up to two hours after stimulation. None required additional medical intervention.

Although long-term safety data in children are unavailable, computer models predict that the peak electric field applied to the brain would range from 0.36 V/m for a small adult head to 0.50 V/m for a pediatric head (76), suggesting a range well below of what has been neurotoxic in preclinical studies (52). Other authors have cautioned against the use of tDCS and other brain stimulation modalities in children and adolescents (77, 78), highlighting the vulnerability of the young brain (79) with sensitive periods during which limited intervention can yield unexpected or detrimental results (80). Davis argues that several questions remain unanswered regarding tDCS in pediatric patients, including the potential unknown effects of stimulation, differential side effects in children, limited evidence to guide dosing parameters, and a dearth of translational studies focused on children and adolescents (77).

While few could disagree with Davis’ critique, the same arguments could be levied against electroconvulsive therapy (ECT), which has been used for decades, albeit circumspectly, in child and adolescent psychiatry. ECT is associated with known effects of memory impairment, prolonged and delayed seizures, headaches, confusion, nausea, muscular pain, and anesthetic risks (81–83). The long-term risks of ECT in adolescents are obscure. Moreover, few randomized controlled trials in adolescents exist to guide its use (84). Nonetheless, ECT is an existing clinical option for treatment-resistant depression in adolescents (84).

Unlike ECT, which induces seizures by definition, or TMS, which provokes seizures rarely (85), tDCS is unlikely to induce seizures. In one study, tDCS of 1 mA for 10 min did not induce any detectable epileptiform activity on EEG in pediatric patients (86). Also unlike ECT, tDCS likely does not significantly impair cognition (53, 87, 88). In fact, researchers have evaluated its potential to enhance attention, learning, and memory (89–93). Early applications of tDCS in children and adolescents with ADHD, childhood-onset schizophrenia, and ASD found that tDCS was well tolerated (56, 61, 94). In one study in childhood-onset schizophrenia, tDCS (2 mA, 20 min) did not induce any detectable changes on MRI, EEG, or ECG (61).

Finally, adult treatments are often clinically adapted for pediatric patients without an evidentiary base in this population. Lithium, for example, was approved by the FDA for the treatment of pediatric bipolar disorder entirely on the basis of data gleaned from bipolar adults. Researchers did not evaluate its actual efficacy in pediatric bipolar patients until years after approval (95). In the late 1980s and 1990s, the use of SSRIs for treating depression in youth also was based largely on extrapolation from adult studies. Although the efficacy of SSRIs for adolescent MDD was later supported in some randomized controlled trials in child and adolescent samples, the long-term effects of SSRIs on the adolescent brain remain obscure (96).

## Conclusion

Existing therapies are inadequate to meet the needs of adolescents with depression. tDCS may offer hope, as available evidence suggests it is safe, tolerable, and acceptable. Furthermore, available data suggest that tDCS is efficacious for depression in adults. Although caution is warranted in the context of neurodevelopment, measured research efforts could develop tDCS as a novel and effective intervention for adolescent depression.

## Author Contributions

All authors (JL, CL, ZJD, and PC) contributed to the conception, structure, and literature review for the manuscript. JL and CL prepared the initial draft, and ZJD and PC critically revised the draft. All authors (JL, CL, ZJD, and PC) prepared the final draft. All authors (JL, CL, ZJD, and PC) approved the final draft for publication and agreed to assume accountability for the accuracy and composition of the manuscript.

## Disclaimer

The content of this review is solely the responsibility of the authors and does not necessarily represent the official views of the National Institutes of Health.

## Conflict of Interest Statement

JL has no disclosures. CL receives research support from the Mayo Clinic Foundation Departmental Small Grant Program and is a site investigator for a multicenter study funded by Neuronetics, Inc. ZJD has received research and equipment in-kind support for an investigator-initiated study through Brainsway Ltd.; he also has served on the advisory board for F. Hoffmann-La Roche Ltd. and Merck & Co., Inc. and has received speaker support from Eli Lilly and Co. PC has received research grant support from Pfizer, Inc., NIMH (K23 MH100266), the Brain and Behavior Research Foundation, and the Mayo Clinic Foundation. He has served as a site subprincipal or principal investigator (without additional compensation) for Eli Lilly and Co., Forest Laboratories, Inc., Merck & Co., Inc., and Pfizer, Inc.; has received equipment support from Neuronetics, Inc.; and receives supplies and genotyping services from Assurex Health, Inc. for an investigator-initiated study. He is a site primary investigator for a multicenter study funded by Neuronetics, Inc.
